# Short communication: Comments on hair disorders associated with dupilumab based on VigiBase

**DOI:** 10.1371/journal.pone.0270906

**Published:** 2022-07-27

**Authors:** Sunny Park, So Hyang Park, Young Joo Byun, Soo An Choi

**Affiliations:** 1 College of Pharmacy and Research Institute of Pharmaceutical Sciences, Korea University, Sejong, South Korea; 2 College of Pharmacy, Korea University, Sejong, South Korea; King Faisal University, SAUDI ARABIA

## Abstract

**Background:**

Dupilumab is a human antibody that blocks the signaling of both interleukin-4 and interleukin-13 receptors. It has been approved for the treatment of moderate-to-severe atopic dermatitis. However, several case reports have reported conflicting effects of dupilumab on alopecia.

**Objectives:**

This study aimed to examine dupilumab-related hair disorders using the large real-world database, VigiBase.

**Methods:**

All individual case safety reports associated with dupilumab in the Uppsala Monitoring Center VigiBase until December 29, 2019, were analyzed. Hair disorder-related terms were defined in High Level Terms with “alopecias,” “pilar disorders NEC (not elsewhere classified),” and “hypertrichoses,” using the Medical Dictionary for Regulatory Activities Hierarchy. Hair disorder reports associated with dupilumab and other biologics that inhibit the Th2 axis (omalizumab, mepolizumab, reslizumab, and benralizumab) were analyzed to determine their association with hair disorders. Disproportionality analysis was performed based on the proportional reporting ratio, reporting odds ratio, and information components.

**Results:**

Among the 20,548 total dupilumab adverse event (AE) reports, hair disorders were reported in 462 dupilumab cases (2.2%), most of which reported hair loss, and only eight cases reported an increase in hair growth. The paradoxical trend in hair loss and growth after dupilumab use was confirmed using a disproportionality analysis. Among the other investigated biologics on Th2 immunity, only omalizumab was associated with hair loss. Additionally, hair disorders after dupilumab treatment were more frequently reported in women than in men. The proportion of hair disorder cases was high in Europe, accounting for 20.8% of hair disorder reports, whereas only 9.7% of all dupilumab-related AEs were reported in Europe. In conclusion, our analysis using a large real-world database confirmed that dupilumab is associated with hair disorders.

## Introduction

Dupilumab is a human antibody that blocks interleukin (IL)-4 and IL-13 receptors, downregulating the type 2 inflammatory pathway [1]. Type 2 inflammation plays a key role in allergic diseases, including moderate-to-severe atopic dermatitis, allergic asthma, allergic rhinitis, and chronic rhinosinusitis with nasal polyps [2–4]. Several biologics that reduce type 2 immunity, including dupilumab, omalizumab, mepolizumab, benralizumab, and reslizumab, have been used to treat allergic diseases [5]. In 2017, dupilumab received the first global approval for the treatment of patients with moderate-to-severe atopic dermatitis (AD) and was granted an extension of indication for the treatment of various allergic diseases, including moderate-to-severe asthma, eosinophilic esophagitis, and chronic rhinosinusitis with nasal polyposis.

Owing to the extension of indications, the use of dupilumab has increased and post-marketing safety reports have accumulated. Real-world post-marketing data provide a large dataset from diverse populations that are not included in clinical trials. Pharmacovigilance studies using real-world data can be informative and enhance regulatory decision-making [6]. As pharmacovigilance database, VigiBase is the World Health Organization Uppsala Monitoring Center (WHO-UMC) global database of individual case safety reports (ICSRs) submitted since 1968.

Recently, dupilmab-related hair disorders have been reported in the real-world [7]. Alopecia areata (AA) is a T-cell-mediated autoimmune hair disease [8–10]. Patients with AA show elevated levels of type 2 cytokines [11] suggesting that Th2 axis suppression may be a therapeutic target for AA [12]. Several case reports have reported conflicting effects of dupilumab on alopecia. A previous study has reported dupilumab-related hair disorders [7] and several paradoxical cases of AA development or improvement following dupilumab therapy have been reported [13–16]. Therefore, exploring the association of dupilumab with hair disorders and comparing it to that of other biologics that inhibit the type 2 immune pathway, using a post-marketing real-world database, can provide a comprehensive understanding of dupilumab-related hair disorders. This study aimed to determine dupilumab-related hair disorders using the global pharmacovigilance database, VigiBase.

## Materials and methods

### Data source and ethical statement

This study used VigiBase which is a large global database of ICSRs that contains over 30 million case reports. To determine dupilumab-related hair disorders, all suspected and interacting ICSRs associated with dupilumab in the UMC VigiBase from 1968 to December 29, 2019 were used. Due to the well-known association between AA and Th2 inflammation, other biologics related to type 2 immunity, such as omalizumab, mepolizumab, reslizumab, and benralizumab, were also collected to compare their association with hair disorders. This study was approved by the Institutional Review Board of Korea University (IRB No.2020–0208), which waived the requirement for informed consent as secondary data were used.

### Disproportionality analysis and signal detection criteria

We used disproportionality analysis with a computerized algorithm to identify hidden patterns in a large database. The WHO defines signals as a possible relationship between an adverse event (AE) and a drug being unknown or incompletely documented previously [17]. For signal detection, a two-by-two contingency table was generated (Table 1).

**Table 1:** Two-by-two contingency table of adverse events (AEs) of the drugs of interest.

Number of reports	Specific adverse events (AEs)	All other AEs
**Drug of interests**	A	B
**All other drugs**	C	D

A: number of reports containing both drugs of interest and specific AEs; B: number of reports containing drug-related AEs of interest but with all other AEs; C: number of reports containing specific AEs but with all other drugs; D: number of reports containing all other drugs and all other AEs.

Disproportionality analysis was performed using the Preferred Terms of Medical Dictionary for Regulatory Activities (MedDRA) to identify dupilumab-related signals using the proportional reporting ratio (PRR) [18], reporting odds ratio (ROR) [19], and information component (IC) based on the Bayesian confidence propagation neural network [20]. The three indices of the calculations and signal detection criteria are listed in Table 2. For events reported at least three times, significant signals were defined as PRR and ROR greater than two, and the lower limit of the 95% credibility interval of IC was greater than zero [21]. Statistical analyses were performed using SAS statistical software (version 9.4; SAS Institute Inc., Cary, NC, USA) and Microsoft Excel (2016).

**Table 2:** Formula and signal detection criteria.

Indices	Definition	Criteria for signal
PRR	{A/(A+B)}/{C/(C+D)}	≥2
ROR	(A/B)/(C/D)	≥2
IC	Log₂P(AE,Drug)/P(AE)P(Drug)	Under limit of 95% CI ≥ 0

PRR, proportional reporting ratio; ROR, reporting odds ratio; IC, information component; AE, adverse event; CI, confidence interval; A: number of reports containing both drugs of interest and specific AEs; B: number of reports containing drug-related AEs of interest but with all other AEs; C: number of reports containing specific AEs but with all other drugs; D: number of reports containing all other drugs and all other AEs.

### Hierarchical analysis

Hierarchical analysis was performed to identify hair disorder-related signals using MedDRA version 23.0. All terms were written according to MedDRA notation. MedDRA has five hierarchy levels: System Organ Class (SOC), High-Level Group Term (HLGT), High-Level Term (HLT), Preferred Term (PT), and Lowest-Level Term (LLT). The five-level hierarchical structure of MedDRA is a key feature that enables accurate analysis of reported AEs. MedDRA hierarchical analysis helps bring together similar medical conditions, leading to spontaneously reported standardized term grouping. Hair disorders were defined as the terms in their HLTs with “alopecias,” “pilar disorders NEC (not elsewhere classified),” and “hypertrichoses.”

## Results

Among the 20,548 total dupilumab AE reports, 462 (2.2%) were associated with hair disorders, with 20 cases reporting two hair disorder terms. Table 3 shows the demographics of dupilumab AE reports. Women were more likely to experience hair disorders than men were after dupilumab treatment (65.2% vs. 23.8%, unknown: 11.0%). In Europe, the proportion of total hair disorder-related cases was higher than that of dupilumab-related AEs, with 20.8% of hair disorders compared to only 9.7% of all dupilumab-related AEs. There were 98 dupilumab-related AEs in the Western Pacific Region; however, no hair disorders were recorded. The proportion of dupilumab indications for AD in hair disorder reports was similar to that of all reports.

**Table 3:** Demographic characteristics of dupilumab-related adverse event reports.

Characteristics		No. of all reports (%) [N = 20,548 reports]	No. of hair disorder* reports (%) [N = 462 reports]
Region	Region of the Americas	18,372 (89.41)	363 (78.57)
	European Region	2,002 (9.74)	96 (20.78)
	Eastern Mediterranean Region	76 (0.37)	3 (0.65)
	Western Pacific Region	98 (0.48)	0 (0)
Age	< 2 years	11 (0.05)	0 (0)
	2–11 years	164 (0.8)	0 (0)
	12–17 years	704 (3.43)	4 (0.87)
	18–44 years	5,220 (25.4)	123 (26.62)
	45–64 years	4,852 (23.61)	109 (23.59)
	65–74 years	1,201 (5.84)	33 (7.14)
	≥ 75 years	629 (3.06)	7 (1.52)
	Unknown	7,767 (37.8)	186 (40.26)
Gender	Female	11,271 (54.85)	301 (65.15)
	Male	7,979 (38.83)	110 (23.81)
	Unknown	1,298 (6.32)	51 (11.04)
Indication****	Atopic dermatitis**	11,502 (48.44)	259 (48.22)
	Unknown	7,448 (31.37)	168 (31.28)
	Eczema***	1521 (6.41)	52 (9.68)
	Asthma	1170 (4.93)	12 (2.23)
	Others	2104 (8.86)	46 (8.57)

* Hair disorders include the terms listed in S1 Table.

** Including “Atopic dermatitis,” “Dermatitis atopic," "Dermatitis allergic," and "Dermatitis atopic aggravated."

***Including "Eczema," and “Atopic Eczema."

**** One report has one or more indications.

Table 4 shows the signals of dupilumab and other biologics detected in the disproportionality analysis based on the MedDRA hierarchy. Although dupilumab was associated with both hair loss and growth, most of the cases reported hair loss whereas only eight cases reported an increase in hair growth. Notably, no pharmacovigilance signal for hair disorders associated with anti-IL-5 biologics (mepolizumab, reslizumab, and benralizumab) was found, even though those have been reported in VigiBase. Our analysis revealed that only omalizumab (anti-IgE) was associated with hair loss but not hair growth.

**Table 4:** Disproportionality analysis results of signals.

Drug	PT	LLT	Number of reports*	PRR	ROR	IC_025_
Dupilumab	Alopecia	Alopecia	377	2.26	2.28	1.02
		Hair loss	50			
		Hair thinning	4			
		Body hair loss	1			
	Alopecia areata	Alopecia areata	38	23.34	23.38	3.66
	Alopecia universalis	Alopecia universalis	3	13.97	13.97	0.24
	Hair growth abnormal	Hair growth abnormal	11	3.65	3.65	1.05
		Hair growth increase	8			
Omalizumab	Alopecia	Alopecia	594	2.92	2.97	1.44
		Hair loss	165			
		Hair thinning	6			
		Accelerated hair loss	4			
		Alopecia reversible	1			
		Atrichosis	1			
	Alopecia areata	Alopecia areata	11	4.21	4.21	0.90

IC_025_: lower limit of the 95% credibility interval for the information component; PT, Preferred Term; LLT, Lowest-Level Term; PRR, proportional reporting ratio; ROR, reporting odds ratio.

* the number of reports was calculated based on the LLT term level, and a case was reported for one or more LLT terms.

## Discussion

In our disproportionality analysis, dupilumab was associated with both hair loss and growth, which is consistent with a previous systematic review based on case reports [7]. Eight case reports have described hair growth after dupilumab use in our analysis as previously reported cases [15, 16, 22–29]. In contrast, our results indicate AA complications as several cases reporting AA onset, reactivation, or exacerbation after dupilumab use [13, 14, 30–40]. Several hypotheses regarding dupilumab-induced AA complications or improvements have been proposed [12, 41, 42]. Some studies have suggested that Th2 inhibition may amplify alternate immune pathways, such as Th1 or Th17 [12, 41], leading to hair loss. In contrast, dupilumab-induced IL-4 inhibition is thought to result in a decrease in inflammatory mediators, potentially leading to concomitant improvement in AA and AD [16].

Among other biologics that inhibit Th2 pathways, only omalizumab was associated with alopecia in this study, probably due to the increased total IgE levels after omalizumab use. Omalizumab, an anti-IgE agent, is known to decrease free IgE levels and increase total IgE levels [43, 44]. Our disproportionality analysis revealed that, unlike omalizumab, dupilumab induced both hair growth and hair loss. A phase 2a trial has suggested that the baseline IgE level is a good predictor of dupilumab efficacy in AA [45], and a previous study has reported high total IgE levels in patients with AA [46]. Dupilumab is considered to have paradoxical effects as a previous study has reported a case of allergic conjunctivitis with IgE reduction and hypereosinophilia after dupilumab treatment [47]. Hypereosinophilia has also been associated with alopecia in a previous study [48]. In addition to the paradoxical effects of dupilumab on IgE and eosinophil levels [47], the long-term effects of dupilumab may vary due to AA progression and the relationship between AA and AD [49]. Moreover, the pathogenesis of alopecia is complex, and different levels of IgE and IL-4 have been reported, depending on the various types of alopecia [50]. Overall, the paradoxical results of dupilumab in hair disorders may be caused by these complex disease factors.

The factors affecting the effects of dupilumab in hair disorders are poorly understood. In our study, all AEs after dupilumab use were predominantly reported in the American region (89.4%), and only a few (9.7%) were reported in the European region. However, the European region has reported a relatively higher proportion of hair disorders (20.8%) after dupilumab use. Additionally, all eight cases of “hair growth increase” were reported in Europe. These results suggest racial differences, which is in alignment with a previous study reporting racial differences in alopecia [51]. Contrary to previous studies showing a greater incidence of alopecia in men after dupilumab treatment [7, 52], our study showed that hair disorders were predominant in women. The pathogenesis and risk factors of alopecia are complex, and various studies have suggested that the overall sex predominance of alopecia is linked to race [53–58]. Therefore, further studies are needed to determine the effects of sex and race on alopecia.

The main limitation of this study is that the effect of the disease cannot be excluded. Atopic background and Th2-skewing are suggested to play a key pathogenic role in AA [45]. A recent clinical trial on the efficacy of dupilumab in patients with AA, with or without AD, has reported a possible pathogenic role of the Th2 axis [45]. In contrast, one case of AD improvement but AA occurrence after dupilumab therapy showed AA improvement but worsened AD after discontinuation of dupilumab, [31] despite the clear association between AA and AD [49]. Although the effects of the disease cannot be completely ruled out, hypereosinophilia was reported after dupilumab use [48] and alopecia was main AE of hair disorders after dupilumab use but not other biologics that inhibit IL-5 in our study, suggesting that hair disorders after dupilumab use cannot be explained by the effects of the disease alone.

Spontaneous safety reports also have limitations such as reporting biases and lack of information, including AD severity, types of alopecia, comorbidities, and prognosis. There may be a reporting bias between hair loss and growth because hair growth is considered a more positive symptom than hair loss. Moreover, it was difficult to evaluate causal relationships because the observations were cross-sectional. Thus, further studies are required to clarify the causal relationship between dupilumab and AA, and a novel therapeutic paradigm for dupilumab in AA. Despite these limitations, signals from post-marketing data can help develop new safety information [59]. This study confirms the paradoxical effects of dupilumab on AA using a real-world database. In conclusion, using a real-world database, VigiBase, we confirmed that dupilumab is associated with hair disorders by disproportionality analysis.

## Supporting information

S1 Table Hair disorder-related adverse event reports according to the medical dictionary for regulatory activities hierarchy. (DOCX)
