# Supplementary material for: Short communication: Comments on hair disorders associated with dupilumab based on VigiBase
Source: PLoS One. 2022 Jul 27;17(7):e0270906. doi: 10.1371/journal.pone.0270906 (PMC9328572; doi:10.1371/journal.pone.0270906)
Supplement: S1 Table — (DOCX) [file pone.0270906.s001.docx]

Table S1 Hair disorder-related adverse event reports of dupilumab according to the Medical Dictionary for Regulatory Activities hierarchy

| **SOC** | **HLGT** | **HLT** | **PT** | **Number of reports** |
| --- | --- | --- | --- | --- |
| Skin and subcutaneous tissue disorders | Skin appendage conditions | Alopecias | Alopecia | 377 |
|  |  |  | Alopecia areata | 38 |
|  |  |  | Alopecia totalis | 3 |
|  |  |  | Alopecia universalis | 3 |
|  |  |  | Diffuse alopecia | 9 |
|  |  |  | Madarosis | 14 |
|  |  | Pilar disorders NEC | Hair disorder | 2 |
|  |  |  | Hair growth abnormal | 19 |
|  |  |  | Trichorrhexis | 4 |
|  |  |  | Hair colour changes | 5 |
|  |  |  | Hair texture abnormal | 4 |
|  |  | Hypertrichoses | Hirsutism | 1 |
|  |  |  | Hypertrichosis | 3 |

NEC, not otherwise classified; SOC, System Organ Class; HLGT, High Level Group Term; HLT, High Level Term; PT, Preferred Term
